# Duplex quantitative real-time PCR assay for the detection and discrimination of the eggs of *Toxocara canis* and *Toxocara cati* (Nematoda, Ascaridoidea) in soil and fecal samples

**DOI:** 10.1186/1756-3305-5-288

**Published:** 2012-12-07

**Authors:** Jean-Francois Durant, Leonid M Irenge, Renata Fogt-Wyrwas, Catherine Dumont, Jean-Pierre Doucet, Bernard Mignon, Bertrand Losson, Jean-Luc Gala

**Affiliations:** 1 Centre de Technologies Moléculaires Appliquées, Institut de Recherche Expérimentale et Clinique, Université catholique de Louvain, Clos chapelle-aux-champs, 30 B1.30.24, 1200, Brussels, Belgium; 2 Defense Laboratories Department, ACOS Ops&Trg, Belgian Armed Forces, Martelarenstraat, 181, 1800, Peutie, Belgium; 3 Department of Biology and Environmental Protection, University School of Physical Education, Królowej Jadwigi 27/39, 61-871, Poznań, Poland; 4 Royal Military Academy, Avenue de la Renaissance 30, 1000, Bruxelles, Belgium; 5 Clinivet, clinique vétérinaire de Gosselies, rue pont à Migneloux 39, 6041, Gosselies, Belgium; 6 Département des Maladies Infectieuses et Parasitaires, Faculté de Médecine Vétérinaire, Université de Liège (Ulg), boulevard de Colonster, 20, B43, 4000, Liège, Belgium

**Keywords:** Duplex real-time PCR, ITS2, Toxocara, Eggs, Fecal, Soil, Samples

## Abstract

**Background:**

Toxocarosis is a zoonotic disease caused by *Toxocara canis* (*T. canis*) and/or *Toxocara cati* (*T. cati*)*,* two worldwide distributed roundworms which are parasites of canids and felids, respectively. Infections of humans occur through ingestion of embryonated eggs of *T. canis* or *T. cati*, when playing with soils contaminated with dogs or cats feces. Accordingly, the assessment of potential contamination of these areas with these roundworms eggs is paramount.

**Methods:**

A duplex quantitative real-time PCR (2qPCR) targeting the ribosomal RNA gene internal transcribed spacer (ITS2) has been developed and used for rapid and specific identification of *T. canis* and *T. cati* eggs in fecal and soil samples. The assay was set up on DNA samples extracted from 53 adult worms including *T. canis*, *T. cati*, *T. leonina*, *Ascaris suum* (*A. suum*) and *Parascaris equorum* (*P. equorum*). The assay was used to assess the presence of *T. cati* eggs in several samples, including 12 clean soil samples spiked with eggs of either *T. cati* or *A. suum*, 10 actual soil samples randomly collected from playgrounds in Brussels, and fecal samples from cats, dogs, and other animals. 2qPCR results on dogs and cats fecal samples were compared with results from microscopic examination.

**Results:**

2qPCR assay allowed specific detection of *T. canis* and *T. cati*, whether adult worms, eggs spiked in soil or fecal samples. The 2qPCR limit of detection (LOD) in spiked soil samples was 2 eggs per g of soil for a turnaround time of 3 hours. A perfect concordance was observed between 2qPCR assay and microscopic examination on dogs and cats feces.

**Conclusion:**

The newly developed 2qPCR assay can be useful for high throughput prospective or retrospective detection of *T.canis* and/or *T. cati* eggs in fecal samples as well as in soil samples from playgrounds, parks and sandpits.

## Background

Toxocarosis is a zoonotic disease caused by the larvae of *Toxocara,* a worldwide distributed roundworm genus of the ascaroid group. *Toxocara* species of human and animal health significance are essentially represented by *T. canis* and *T. cati*, parasites of canids and felids, respectively [1]. According to recent data, the prevalence of *T. canis* or *T. cati* is variable but remains high [2]. This widespread prevalence of *Toxocara* spp. in dogs and cats is associated with the contamination of playgrounds, municipal parks and households with eggs [3-5]. Red foxes (*Vulpes vulpes*) are also frequently infected by *T. canis,* an observation to consider in the light of recent epidemiological studies, which point out the progressive increase in the number of foxes in European urban environments over the last few years [6,7]. Children are most likely infected through ingestion of embryonated eggs of *T. canis* or *T. cati* when playing on soils contaminated with dogs or cats feces containing *Toxocara* eggs. After ingestion, *Toxocara* eggs hatch in intestine and release larvae (juvenile worms) that penetrate the small intestine wall to enter the bloodstream. They subsequently travel through the bloodstream to all the major organs. Although most infections are asymptomatic, two well-defined syndromes are classically recognized in humans: visceral larva migrans (VLM), a systemic disease caused by larval migration through major organs, and ocular larva migrans (OLM), a disease limited to the eye and optic nerve. Less severe syndromes have been described, in children (covert toxocariasis) and in adults (common toxocariasis) [8-10].

Accordingly, monitoring the presence of *Toxocara* eggs in dogs and cats feces, as well as in playgrounds and municipal parks likely to be contaminated by animal stools is critical in the control of toxocarosis.

Microscopic examination of dog and cat stools or soil samples is commonly used for identification of *Toxocara* eggs. The method includes an enrichment pre-analytical step through the use of centrifuge-flotation techniques [11]. However, the method displays poor sensitivity, due to the low recovery of *Toxocara* eggs especially from soil samples. Furthermore, microscopic examination can fail to unambiguously discriminate eggs of *Toxocara* species because they are fairly similar [12,13]. Other important roundworms include *Baylisascaris procyonis*, the common intestinal roundworm of raccoons responsible for a severe human neurologic disease [14] and possibly *Toxocara vitulorum* (*T. vitulorum*), a cattle roundworm, which has been linked to a low level zoonosis alleged to affect children. There is, however, much uncertainty about the zoonotic potential of this species, as infections attributed to *T. vitulorum* could be due to *T. canis* or *T. cati*[2]. Owing to their high sensitivity and high specificity, PCR methods have been highlighted to improve the detection and identification of *Toxocara* species of human significance. Numerous PCR methods for the detection and identification of *T. canis* and *T. cati* were reported to identify *T. canis* and *T. cati* in dog, fox and cat stools [15], as well as in soil samples [16,17]. The DNA-based methods take advantage of the high genetic variability within molecular markers such as ITS2 for the discrimination of *T. canis* and *T. cati* from their closely-related neighbors, namely *T. leonina*, *T. vitulorum* and *T. malaysiensis*[2,17]. However, despite these achievements, many drawbacks actually preclude their implementation in routine screening of *Toxocara spp* of clinical significance, either in dog, cat and fox stools or in soil samples from playgrounds and parks. These pitfalls include the risk of carry over contamination, the low throughput of samples analysis, the difficulty of automation and the lack of standardization. Accordingly, real-time PCR has the potential to circumvent the drawbacks of endpoint PCR. Moreover, real-time PCR is rapid and can allow analysis of many samples in a short time without any need of additional post-PCR manipulations, often responsible for carry-over contamination. Consequently, the development of a real-time PCR assay for detection of *Toxocara* eggs could improve diagnosis of toxocariosis and thus improve health status of children in contaminated areas.

In the current study, we have developed a 2qPCR assay for rapid and specific identification of *T. canis* and *T. cati* eggs in fecal samples as well as in soil samples from sandpits and playgrounds. Results suggest that the 2qPCR assay is sensitive and specific for detecting *T. canis* and/or *T. cati* both in fecal and soil samples.

## Methods

### Biological and environmental samples

Three adult worms (one representative each from *T. canis*, *A. suum* and *P. equorum)*, were provided by the Parasitology unit of the Faculty of Veterinary Medicine (FMV) of the University of Liège and subsequently used throughout the study as positive and negative controls. Fifty worms previously identified as *T. canis* (n = 30), *T. cati* (n = 14) or *T. leonina* (n = 6) by macroscopic and microscopic examination were kindly provided by Dr. R. Fogt (Department of Biology and Environmental Protection, University School of Physical Education, Poznań, Poland). Egg suspensions of *T. cati* and *A. suum* recovered from two adult worms were provided by FMV.

### Microscopic observations

Suspensions of *T. cati* and *A. suum* eggs were kindly provided by B. Mignon. For egg quantification, smears of 20 μL of egg suspension were observed under light microscopy and counted. This process was performed in triplicate and the number of eggs in suspension was calculated as the mean from the three counts.

Microscopic examination for *Toxocara* egg identification was performed on enriched fecal samples through the flotation technique, as described previously [11].

### Spiking of soil samples with *T. cati* or *A. suum* eggs

Twelve control soil samples, each made of 5 g of clean sand were spiked with known amounts of *T. cati* eggs (from 100 ± 30 to 5 ± 2) or *A. suum* eggs (from 1020 ± 280 to 5 ± 2).

### Molecular analysis

#### DNA extraction

DNA extraction was carried out on a suspension containing known amounts of *T. cati* and *A. suum* eggs. Briefly, the eggs in suspension were incubated with 100 μL of buffer G2 (Qiagen, Hilden, Germany) and with 20 μL of proteinase K (Qiagen, Hilden, Germany) at 56°C during 2 hours. DNA was purified with BioRobot EZ1 (Qiagen, Hilden, Germany), using a DNA tissue kit, according to the manufacturer's instructions. The DNA was finally eluted in 100 μL of buffer and stored at −20°C until use.

DNA was extracted from 53 adult worms. Briefly, a piece of ~0.2 cm long was cut from each worm, minced with a scalpel blade on a sterile glass slide, and re-suspended in 500 μL NucliSENS® lysis buffer (NucliSENS® lysis magnetic extraction reagents, NucliSENS® miniMAG System, Biomérieux bv, Boxtel NL). The DNA was extracted from the suspension using the NucliSENS® miniMAG system and reagents according to the manufacturer’s instructions. DNA was eluted in 100 μL and stored at −20°C until use.

The QIAamp® DNA Stool (Qiagen®, Leusden, The Netherlands) commercial kit was used to extract DNA from 45 animal fecal samples. In total ~250 mg of each fecal sample was processed according to the recommendations of the manufacturer. DNA solutions were eluted in 200 μL of buffer and stored at −20°C.

Total DNA was extracted from 5 g of soil samples (both spiked and actual soils) using the PowerMax® soil DNA isolation kit and re-suspended in a final volume of 5mL of elution buffer as recommended by the manufacturer. The 5 mL of DNA extract solution was then reduced to 50 μL following an ethanol precipitation protocol recommended by the PowerMax® soil DNA isolation kit manufacturer and stored at −20°C until use.

#### The 2qPCR real-time assay

Internal transcribed spacer 2 (ITS2) was selected as a target for the amplification the *T. canis* and *T. cati.* Briefly, the 2qPCR amplification was performed to specifically identify *T. canis* or *T. cati*. Primers and probes were designed manually in the *T. canis* and *T. cati*-specific part of ITS2 after multiple-alignment of the following ITS2 sequences: *T. canis* [Genbank: AB110034], *T. cati* [Genbank: AB110033], *T. leonina* [Genbank:Y09490], *T. vitulorum* [Genbank:EU189085] and *T. malaysiensis* [Genbank:AM231609]. ITS2 duplex- amplification was based on the use of two forward primers specific for *T. canis* (5’-GCGCCAATTTATGGAATGTGAT-3’) and *T. cati* (5’-ACGCGTACGTATGGAATGTGCT-3’) respectively, and a consensus reverse primer common to both species (5’-GAGCAAACGACAGCSATTTCTT-3’). Moreover, the 2qPCR use two specific probes targeting *T. canis* (5’-FAM-CCATTACCACACCAGCATAGCTCACCGA-3’-BHQ1) and *T. cati* (5’-Cy5-TCTTTCGCAACGTGCATTCGGTGA-3’-BHQ3). The selected primer candidates and the probes were tested *in silico* against all the publicly available nucleotide sequence databases by using BLASTN [18]. The expected amplicon sizes for *T. canis* and *T. cati* were 141-bp and 155-bp*,* respectively. Primers and probes were purchased from Eurogentec (Ougrée, Belgium).

Each 2qPCR was carried out in 25 μL of a reaction mixture containing 2.5 μL of extracted DNA as template, 300 nM of each primer, 100 nM of each probe and 12.5 μL of LightCycler® 480 Probes Master 2x (Roche Diagnostics GmbH, Mannheim, Germany). Amplification was performed on a Roche LightCycler® 480 System Real-Time PCR system (Roche Diagnostics GmbH, Mannheim, Germany). The reaction was initiated at 95°C for 5 min followed by 45 cycles of denaturation at 95°C for 10 s, annealing at 60°C for 15 s and extension at 72°C for 5 s. Each sample was tested in triplicate and data were recorded as crossing points (Cq) on a Roche LightCycler® 480 System, using the analytical software LCS480 1.2.9.11 from the same manufacturer.

Standard curves were constructed from serial dilutions of *T. canis* and *T. cati* DNA. Cq values obtained by 2qPCR assay were plotted against the logarithm of DNA amount to assess the dynamic range.

A sample was considered as positive when all wells within the triplicate were associated with an exponential fluorescence with a Cq value <40.00. The specificity of the test was investigated by performing the 2qPCR analysis on DNA samples from worms (n =53) and negative controls from fungal and bacterial DNA (n = 33) and human DNA (n = 16). In order to define the LOD, a standard curve was constructed of 10:10 serially diluted DNA of these 2 DNA solutions which were used as a template for 2qPCR assays. Cq values obtained were plotted against the logarithm of copies to assess the dynamic range. The efficiency of 2qPCR assays was calculated as described by Wong and Medrano [19].

#### In-house qPCR assay

An *in*-house qPCR previously designed at CTMA and targeting the conserved region of the 18S rRNA in a wide range of Ascaridoidea was carried out on DNA batches extracted from all samples used in this study. This PCR was used as an internal quality control for DNA extraction while being also used to detect the presence of Ascaridoidea in *T. canis* and *T. cati*-negative soil or fecal samples.

Primers and the probe were designed manually after a multiple alignment of the 18S sequences including *T. canis* [Genbank:U94382)], *T. cati* [Genbank:AF480059], *T. leonina* [Genbank:U94383], *T. vitulorum* [Genbank:EF180078], *A. suum* [Genbank:AF036587] and *P. equorum* [Genbank:U94378]. The 18S rRNA amplification was based on the use of a pair of primers (primer forward: 5’-CTACCACATCCAAGGAAGGCA-3’; primer reverse: 5’-TTATTTTTCGTCACTACCTCCTCATG-3’) and a probe (5’-CAGGCGCGCAAATTACCCACTCTC-3’) labeled by the tandem Reporter-Quencher Red610-BHQ2.

The specificity of the assay was further tested on several fecal samples from animals other than cats and dogs. These included randomly collected fecal samples from calves and/or cows (n = 9), horses (n = 2), rabbits (n = 3), hens (n = 4) as well as a single fecal sample from a donkey, a pig and a pigeon (Table 1). Noteworthy, these animals are not hosts of *T. canis* and *T. cati*, though they are common hosts of other roundworms species. For each negative sample, PCR inhibition was assessed as previously described [20]. This process also included the testing of a panel of DNA from bacteria (n = 25) and fungi (n = 8) (Table 2) and human DNA (n = 16).

**Table 1:** 2qPCR results from fecal samples animals other than dogs and cats

**Animals Origins**	**N**	**Macro- micro-scopic observation**	***T. canis *FAM Cq value**	***T. cati *Cy5 Cq value**	***In*-house qPCR Red610 Cq value**	***T. canis */ *T. cati *molecular identification**	**References**
Calf	1	ND	> 40.00	> 40.00	36.81 ± 0.24	Negative	CAL016
Calves	7	ND	> 40.00	> 40.00	> 40.00	Negative	CAL140,176,107,112,19,26,79
Rabbit	1	ND	> 40.00	> 40.00	36.73 ± 0.52	Negative*	NEM003
Rabbits	2	ND	> 40.00	> 40.00	> 40.00	Negative	NEM009,014
Hen	1	ND	> 40.00	> 40.00	35.44 ± 0.49	Negative*	NEM013A
Hen	1	ND	> 40.00	> 40.00	35.81 ± 0.24	Negative*	NEM013B
Hen	1	ND	> 40.00	> 40.00	33.30 ± 1.54	Negative*	NEM023A
Hen	1	ND	> 40.00	> 40.00	33.88 ± 0.63	Negative*	NEM023B
Cow	1	ND	> 40.00	> 40.00	> 40.00	Negative	NEM015
Pigeon	1	ND	> 40.00	> 40.00	36.20 ± 1.09	Negative*	NEM016
Horses	2	ND	> 40.00	> 40.00	> 40.00	Negative	NEM024,025
Donkey	1	ND	> 40.00	> 40.00	> 40.00	Negative	NEM017
Pig	1	ND	> 40.00	> 40.00	> 40.00	Negative	NEM018

* These samples displayed a positive signal with the *in*-house PCR, suggesting that they harbored other roundworms eggs.

**Table 2:** Bacterial and fungal DNA used for the setting up of the 2qPCR assay

***Species***	**Strain and isolate references**
*Bacillus anthracis*	CEB 9434
*Bacillus cereus*	DSM 345
*Enterococcus casseliflavus*	DSM 10255
*Staphylococcus aureus*	ATCC 35884
*Streptococcus oralis*	DSM 20627
*Streptococcus pneumoniae*	ATCC 6314-D
*Streptococcus pyogenes*	ATCC 12344-D
*Acinetobacter calcoaceticus*	DSM 30006
*Citrobacter freundii*	DSM 30039
*Escherichia coli*	R453
*Escherichia coli*	R456
*Escherichia coli*	R457
*Haemophilus influenzae*	DSM 4690
*Klebsiella oxytoca*	ATCC 700324-D
*Klebsiella pneumoniae*	ATCC 700721-D
*Legionella pneumophila*	DSM 7513
*Moraxella catarrhalis*	DSM 9143
*Neisseria gonorrhoeae*	ATCC 53420-D
*Neisseria meningitidis*	DSM 10036
*Providencia stuartii*	DSM 4539
*Pseudomonas aeruginosa*	DSM 50063
*Pseudomonas fluorescens*	DSM 50090
*Pseudomonas syringae*	DSM 1241
*Serratia marcescens*	DSM 30121
*Stenotrophomonas maltophilia*	DSM 8573
*Alternaria alternata*	CTMA 07-022
*Aspergillus fumigatus*	CTMA 07-035
*Botrytis cinerea*	CTMA BC/07/31
*Cladosporium cladosporoides*	CTMA 07-019
*Cladosporium herbarum*	CTMA CH/07/41
*Epicoccum nigrum*	CTMA 07-086
*Pleospora herbarum*	CTMA 07-077
*Trichophyton rubrum*	CTMA MYC001

ATCC: American Type Culture Collection.

CEB: Centre d’Etude du Bouchet, Vert le Petit, France.

DSM: Deutsche Sammlung von Mikroorganismen und Zellkulturen GmbH.

CTMA: Center for Applied Molecular Technologies, private DNA sample collection.

The test phase for the presence of *T. canis* and *T. cati* in fecal samples was carried out on a panel of 24 feces from dogs (n = 14) and from cats (n = 10) collected by the Clinivet veterinary centre (Table 3). Molecular results were compared with results of the light microscopic examination when these were available.

**Table 3:** 2qPCR results from dog and cat fecal samples

**Animals Origins**	**N**	**Macro- micro-scopic observation**	***T. canis *FAM Cq value**	***T. cati *Cy5 Cq value**	***In*-house qPCR Red610 Cq value**	***T. canis */ *T. cati *molecular identification**	**References**
Dogs	9	Negative	> 40.00	> 40.00	> 40.00	Negative	CN4,5,6,7,8,10,12,14,20
Dog	1	Negative	> 40.00	> 40.00	36.76 ± 0.08	Negative	CN1
Dog	1	Negative	> 40.00	> 40.00	35.86 ± 0.15	Negative	CN2
Dog	1	Negative	> 40.00	> 40.00	37.97 ± 0.15	Negative	CN9
Dog	1	Negative	> 40.00	> 40.00	36.27 ± 0.22	Negative	CN11
Dog	1	Negative	> 40.00	> 40.00	35.67 ± 0.92	Negative	CN13
Cats	7	Negative	> 40.00	> 40.00	> 40.00	Negative	CT1,3,4,5,6,7,8
Cat	1	*Toxocara* eggs and worms	> 40.00	34.68 ± 0.82	35.68 ± 0.24	*Toxocara cati*	CT2
Cat	1	*Toxocara* eggs	> 40.00	35.07 ± 0.55	34.00 ± 0.51	*Toxocara cati*	CT9
Cat	1	*Toxocara* eggs	> 40.00	36.28 ± 0.67	36.75 ± 0.20	*Toxocara cati*	CT10

The 2qPCR assay was also used to assess the presence of *T. canis* and/or *T. cati* in 10 soil samples collected from sandpits and playgrounds across various areas of Brussels city (Belgium).

### Statistical analysis

Statistical analyses were performed using the SPSS statistical package release 12.0 for Windows (SPSS, Inc., Chicago, IL). Concordance between 2qPCR and microscopic observation was calculated using the Kappa statistics of Cohen, to assess the degree of agreement between these different methods.

## Results

### Limit of detection of *T. canis* and *T. cati* DNA by 2qPCR

**Figure 1:**
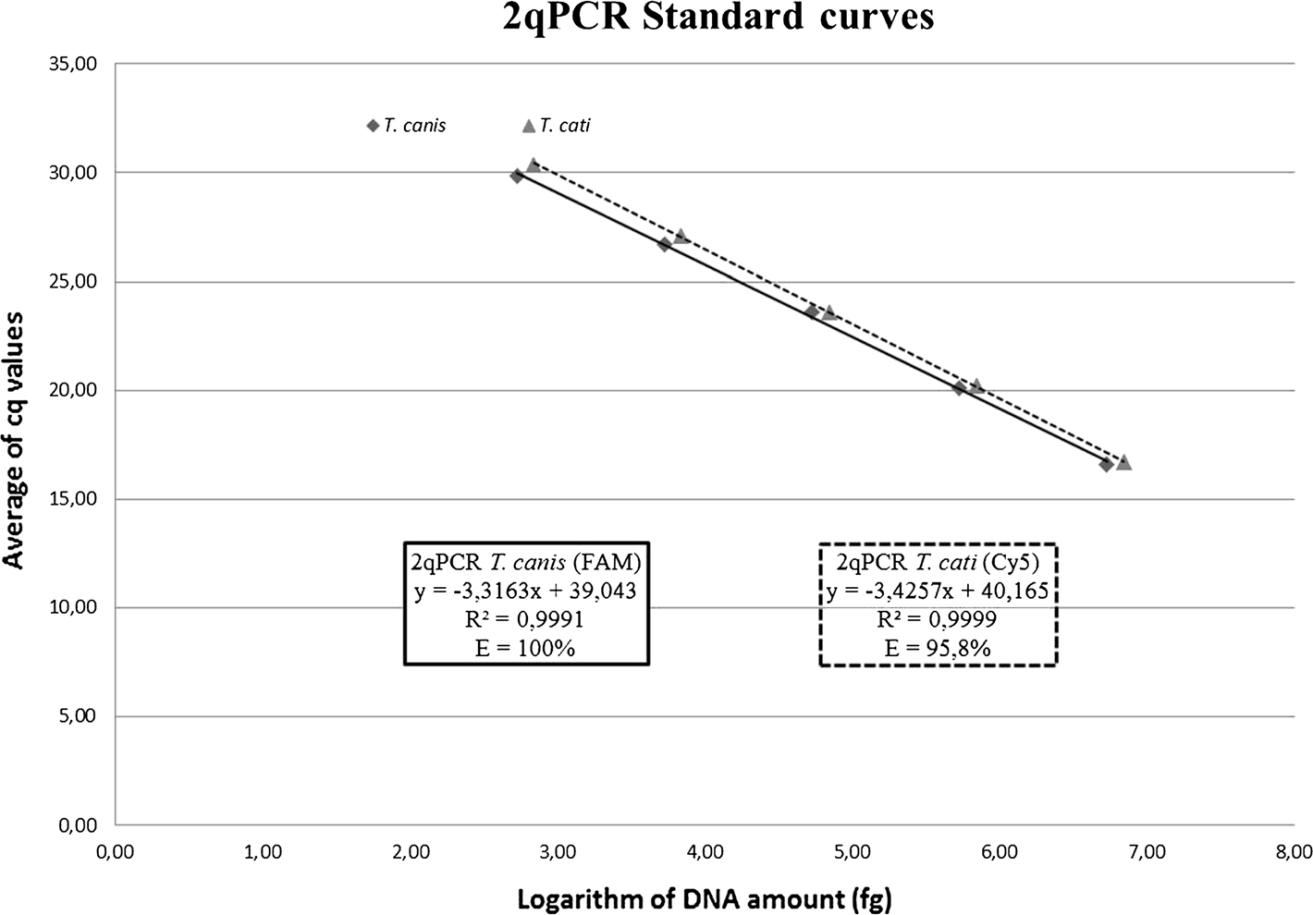
**2qPCR standard dilution curves for *Toxocara canis *and *Toxocara cati. *** Mean Cq values (Y axis) plotted against logarithm of DNA amount used for amplification (X axis). Continuous line represents the dilution curve for *T. canis* 2qPCR amplification whereas the dashed line corresponds to the dilution curve of *T. cati* 2qPCR amplification. E = PCR efficiency; R² = Square of linear correlation coefficient.

The PCR efficiencies were 100% and 95.8% for *T. canis* and *T. cati*, respectively (Figure 1). Significant and reproducible fluorescence signals generated by *T*. *cati* or by *A. suum* were consistently obtained with a DNA solution equivalent to 2 eggs per g of soil (Table 4). Regarding *T. canis*, the LOD estimation was based on DNA extracted from adult worms and calculated at 10 fg per assay. Noteworthy, prior to these experiments, the potential contamination of the soil sample to be spiked with Ascaridoidea eggs was ruled out by performing the 2qPCR as well as the *in*-house qPCR on these 12 soil samples.

**Table 4:** **2qPCR and *in *-house qPCR assays on soil samples spiked with *T. cati *and/or *A. suum *eggs**

**Samples**	**Eggs origins**	**Amount of eggs Mean ± SD**	***T. canis *Cq FAM Mean ± SD**	***T. cati *Cq Cy5 Mean ± SD**	***In*-house Cq Red610 Mean ± SD**
E25	*T. cati*	100 ± 30	> 40.00	25.18 ± 0.06	25.83 ± 0.05
E26	*T. cati*	100 ± 30	> 40.00	25.29 ± 0.02	25.97 ± 0.03
E27	*T. cati*	10 ± 3	> 40.00	31.12 ± 1.03	32.00 ± 0.76
E28	*T. cati*	10 ± 3	> 40.00	31.49 ± 0.53	29.53 ± 0.77
E29	*T. cati*	5 ± 2	> 40.00	30.62 ± 0.84	31.49 ± 0.53
E30	*T. cati*	5 ± 2	> 40.00	> 40.00	> 40.00
E31	*T. cati*	5 ± 2	> 40.00	30.13 ± 1.01	31.10 ± 0.75
E9	*A. suum*	1020 ± 280	> 40.00	> 40.00	25.93 ± 0.06
E10	*A. suum*	102 ± 28	> 40.00	> 40.00	30.08 ± 0.22
E32	*A. suum*	102 ± 28	> 40.00	> 40.00	29.66 ± 0.15
E11	*A. suum*	10 ± 3	> 40.00	> 40.00	33.70 ± 1.47
E12	*A. suum*	5 ± 2	> 40.00	> 40.00	> 40.00
E13	*No egg spiked*	0	> 40.00	> 40.00	> 40.00

Cq values higher than 40.00 are considered as negative results.

### Assessment of the specificity of the 2qPCR

The 2qPCR assay generated specific 6-FAM fluorescence signals with all DNA samples extracted from *T. canis* adult worms (n = 31), whereas DNA samples extracted from *T. cati* adult worms (n = 14) or eggs (n = 2) generated specific Cy-5 signals. No single *T. cati* DNA sample generated 6-FAM fluorescence, nor did any *T. canis* sample generate Cy-5 fluorescence. Sequence analysis of each amplified target confirmed a 100% identity with the corresponding worm-ITS2 molecular targets. The 2qPCR also remained negative with DNA from *A. suum* (one adult worm and eggs solutions), from *P. equorum* (1 adult worm) and from *T. leonina* (6 adult worms). The assay also remained negative with human DNA as well as all bacterial DNA tested. No PCR inhibition was observed when assaying both fecal and soil samples.

### Test phase on fecal samples

From the 24 cat and dog fecal samples examined (Table 3), three cat samples (CT2, CT9 and CT10) displayed real-time PCR signals consistent with the presence of *T. cati*. In each of these samples, *T. cati* eggs were visualized by microscopic examination. In addition, adult worms could be seen in feces CT2. The 14 feces samples from dogs (Table 3) remained negative as also were the 21 feces samples from other animals (Table 1). Nonetheless, while these samples were negative for *T. canis* and *T. cati*, several of them, (CAL016, NEM003, NEM013A, NEM013B, NEM023A, NEM023B, and NEM016) displayed a positive signal with the *in*-house qPCR (suggesting the presence of non-*T. cati/canis* Ascaridoidea eggs in these samples). Results of the 2qPCR assay and microscopic examination on the 24 cat and dog fecal samples were identical. Sensitivity and specificity of the assay were calculated in comparison with microscopic examination (considered as gold standard) on the 24 cat and dog fecal samples and both displayed a 100% value. The kappa score of Cohen, a measure of agreement between microscopic observation and 2qPCR, was 100%.

### Applicability of the assay assessed in actual soil samples

The assay has been used to assess 10 soil samples from sandpits and playgrounds collected in different areas of Brussels city (Belgium). While all the 10 samples remained negative for *T. cati* and *T. canis*, 6 out of 10 samples displayed positive signals with the *in*-house qPCR (Table 5), thus suggesting the presence of non-*Toxocara* Ascaridoidea eggs in these samples. The latter results as well as the previous ones from spiking *Toxocara* eggs in soil show that the assay is applicable for monitoring of the presence of *Toxocara* eggs in soil samples.

**Table 5:** Molecular assays on actual soil samples collected from playgrounds and sandpits

**Soil samples**	***T. canis Cq FAM *Mean ± SD**	***T. cati *Cq Cy5 Mean ± SD**	***In*-house Cq Red610 Mean ± SD**
E33	>40.00	>40.00	30.71 ± 1.44
E34	>40.00	>40.00	29.62 ± 0.03
E17	>40.00	>40.00	>40.00
E18	>40.00	>40.00	>40.00
E19	>40.00	>40.00	32.61 ± 0.40
E20	>40.00	>40.00	31.39 ± 0.25
E21	>40.00	>40.00	32.39 ± 0.37
E22	>40.00	>40.00	>40.00
E23	>40.00	>40.00	27.97 ± 0.16
E24	>40.00	>40.00	>40.00

The samples E33, E34, E19, E20, E21 and E23 are negative for *T. canis* and *T. cati* eggs and positive for non-*Toxocara* Ascaridoidea, whereas samples E17, E18, E22 and E24 are negative for any Ascaridoidea.

## Discussion

We report here the development of a sensitive and specific 2qPCR assay allowing rapid and reliable identification of eggs of *T. canis* and *T. cati* in clinical and environmental samples*.* Increasing populations of dogs, cats and foxes in the urban areas prompt indeed the need for standardized and high throughput analytical methods for studying the prevalence of *Toxocara spp* eggs, mainly *T. canis* and *T. cati*, in fecal and environmental samples [9,17,21,22]. Over the last decade, several methods have been reported for the identification of *Toxocara spp* in environmental samples. These include light and scanning electron microscopy [13] and molecular identification approaches for genotyping and identifying *Toxocara spp*. [15,16].

The aim of this study was to exploit the existing knowledge and expertise in the field of real-time PCR and semi DNA extraction methods for the development of a high throughput method for the identification of *Toxocara spp* in fecal and soil samples. The analysis of stool samples both by 2qPCR and microscopic observation showed a perfect correlation. As illustrated by the current molecular results, combining DNA extraction and 2qPCR contributes to a better standardization regarding the pre-analytical and analytical steps while allowing swift identification of *T. canis* or *T. cati* eggs. Compared with other conventional assays, the real-time PCR technology appears as a high throughput method for detecting *Toxocara* eggs in fecal and soil samples. Accordingly, this molecular assay can be used to assess the contamination of parks, playgrounds and sandpits with eggs of *T. canis* or *T. cati*. In the current study 5 g of soil was directly processed in the assay, yielding a LOD of 2 Ascaridoidea eggs per assay. As highlighted in this work, direct processing of 5 to 10 g of soil followed by a DNA concentration process significantly improves the detection threshold in soil samples while avoiding the use of cumbersome techniques for soil enrichment. Nevertheless, since only a limited number of samples were processed and considering that these samples were predominantly negative, a validation on a large panel of potentially contaminated soil samples could help to confirm the usefulness of the 2qPCR. Our results also showed that animal feces (hens, pigeons, rabbits and calves) and soil samples, presumably harbored worms other than *T. canis* and *T. cati*[23]. This is in line with our positive *in*-house qPCR but negative 2qPCR results, hence confirming the specificity of our real-time PCR assay. Noteworthy, pigeon and rabbit feces are common in municipal parks and other play grounds, along with cat and dog feces. It should also be stressed that while the *in*-house qPCR was used as a simplex real-time PCR, it can be easily scaled up to the 2qPCR and thus constitute a triplex real-time PCR without any impact on the sensitivity of the assay, provided that the real-time PCR platform used is adapted to multiplexing.

The 2qPCR assay appears, therefore, to be a specific, sensitive and reliable tool for identifying *T. canis* and *T. cati* and discriminating them, a result that may be easily overlooked when using microscopic light examination [13]. Additionally, the 2qPCR assay may help discriminating clinically relevant and non-relevant *Toxocara spp* eggs.

Although microscopic examination gave identical results to our 2qPCR assay, the identification process was all but a straightforward process. Eggs from frozen feces were particularly difficult to identify, owing to morphological modifications, and prompting reliance on a highly trained operator. It is of note that none of our soil samples were examined by light microscopy, but one can predict that observation of *Toxocara spp* eggs in this type of sample would have been even more challenging.

Lastly, though not assessed during this work, the assay is also expected to achieve accurate identification of *Toxocara spp* in tissue *larva migrans.* It has been reported indeed that during *larva migrans*, *Toxocara spp* larvae undergo morphological modifications which make species morphological-based identification nearly impossible [24].

## Conclusion

In the present study, a molecular method was developed for allowing a reliable surveillance of fecal and soil sample contamination with eggs of *T. canis* and *T. cati*. Compared to the conventional microscopic examination, used as gold standard, the real-time PCR approach appears to be rapid, displays a high throughput processing rate, while achieving a sensitivity equivalent to the gold standard. Therefore, the current 2qPCR assay appears to be a very promising tool for assessment of contaminated sandpits and playgrounds by *Toxocara spp* eggs.

## Competing interests

The authors report no conflicts of interest. The authors alone are responsible for the content and writing of the paper.

## Authors’ contributions

DJF, ILM and GJL conceived the study; FWR, DJP, MB and LB provided worms, eggs suspensions and fecal samples, DJF, DC and MB carried out microscopic examination. DJF, DC and ILM carried out molecular analyses. ILM and GJL wrote the first draft of the paper, and all authors contributed to the final manuscript which they approve.
